# The Impact of Educational Training for Supporting Chinese American Family Caregivers

**DOI:** 10.1093/geroni/igaa057.1445

**Published:** 2020-12-16

**Authors:** Man Wai Lun

**Affiliations:** Borough of Manhattan Community College, new york city, New York, United States

## Abstract

A pilot self-care educational training, focusing on common caregiving stress, self-care, conflicts, and communication within families for supporting caregiving for family members of old age, was offered to the Chinese American community. After the series of training, 16 participants were asked to evaluate their knowledge of family caregiving as well as the training. Results of the preliminary study revealed that most participants found the training informative but were not sure if they would continue to apply over time. Overall participants reported to be satisfied with the training and receptive to additional training in the future. Results encourage further implementing the training, and investigation of the longitudinal effect of the training to help with family caregiving issues.

